# Accelerating the reconstruction of genome-scale metabolic networks

**DOI:** 10.1186/1471-2105-7-296

**Published:** 2006-06-13

**Authors:** Richard A Notebaart, Frank HJ van Enckevort, Christof Francke, Roland J Siezen, Bas Teusink

**Affiliations:** 1 Center for Molecular and Biomolecular Informatics, Radboud University Nijmegen, P.O.Box 9010, 6500GL Nijmegen, The Netherlands; 2 NIZO food research BV, P.O.Box 20, 6710BA, Ede, The Netherlands; 3 Wageningen Center for Food Sciences, P.O.Box 557, 6700AN Wageningen, The Netherlands; 4 Present address: Friesland Foods Corporate Research, Deventer, The Netherlands

## Abstract

**Background:**

The genomic information of a species allows for the genome-scale reconstruction of its metabolic capacity. Such a metabolic reconstruction gives support to metabolic engineering, but also to integrative bioinformatics and visualization. Sequence-based automatic reconstructions require extensive manual curation, which can be very time-consuming. Therefore, we present a method to accelerate the time-consuming process of network reconstruction for a query species. The method exploits the availability of well-curated metabolic networks and uses high-resolution predictions of gene equivalency between species, allowing the transfer of gene-reaction associations from curated networks.

**Results:**

We have evaluated the method using *Lactococcus lactis *IL1403, for which a genome-scale metabolic network was published recently. We recovered most of the gene-reaction associations (i.e. 74 – 85%) which are incorporated in the published network. Moreover, we predicted over 200 additional genes to be associated to reactions, including genes with unknown function, genes for transporters and genes with specific metabolic reactions, which are good candidates for an extension to the previously published network. In a comparison of our developed method with the well-established approach Pathologic, we predicted 186 additional genes to be associated to reactions. We also predicted a relatively high number of complete conserved protein complexes, which are derived from curated metabolic networks, illustrating the potential predictive power of our method for protein complexes.

**Conclusion:**

We show that our methodology can be applied to accelerate the reconstruction of genome-scale metabolic networks by taking optimal advantage of existing, manually curated networks. As orthology detection is the first step in the method, only the translated open reading frames (ORFs) of a newly sequenced genome are necessary to reconstruct a metabolic network. When more manually curated metabolic networks will become available in the near future, the usefulness of our method in network prediction is likely to increase.

## Background

In recent years, genome sequencing projects have enormously increased our molecular understanding of biological capabilities of organisms. For many research areas, such as biotechnology and biomedical research, the metabolic capacities of cells are highly relevant. On the basis of the functional annotation of predicted genes, genome-scale metabolic networks can be reconstructed [1-3]. An increasing collection of methods is available to analyze the properties of these networks, both from a graph-theoretical point of view [4-7] as well as from a metabolic engineering point of view (for reviews [8] and [9]). Genome-scale metabolic networks are also increasingly being used in integrative bioinformatics [10,11]. As a metabolic network consists of associations between genes and metabolic reactions, it can be used to study the cellular properties of the organism by integrating functional genomics data, such as gene expression and proteomics (the goal of systems biology). The importance of integration has recently been shown in various studies on the topology, dynamics and evolution of metabolic networks [12-14].

Unfortunately, the reconstruction process can be very time-consuming. To a certain extent the effort depends on the level of detail that is required for the purpose of the reconstruction. A metabolic network can be used as an encyclopedia in order to present enzymes in their metabolic context [2]. In this particular case it is not essential to include the exact reaction stoichiometry to analyze functional genomics data (e.g. gene regulation). Extensively curated metabolic encyclopedias can be found in the so-called BioCyc collection, including EcoCyc and the recently developed LacplantCyc [15-17]. On the other hand, the reaction stoichiometry is important for most quantitative modeling purposes, such as flux balance analysis (FBA), in which constraints are defined by the stoichiometry and are used to optimize for a certain flux (e.g. biomass production; for review [8]). In the past few years, several of such metabolic networks for modeling purposes have been constructed [1,18-22].

The metabolic network reconstruction procedure usually starts with functional annotation of genes encoded on the genome of a certain species of interest (reviewed by [2]). Functional annotation is mainly done by homology searches against protein/enzyme databases [23-25]. Several computational methods, such as Pathologic (part of Pathway Tools), have been developed to automate the reconstruction procedure by linking reactions and pathways to the annotated genes (also referred to as gene-reaction associations) [26,27]. As a consequence, the quality of such an automatically generated metabolic network will highly depend on the quality of the annotation. Recently, methods have also been developed to automate the functional annotation and network reconstruction for unannotated genomes [28,29]. In spite of the promising work on developing computational methods to automate the reconstruction of (genome-scale) metabolic networks, in all cases manual curation is still needed, which is the most time-consuming part [1,2].

Incomplete EC-codes and annotation errors in protein databases are likely to be the main cause of errors that arise from homology based computational methods [2,30]. It is, therefore, important to correct these errors by manual investigation of the predictions. Pathologic, for example, is specifically designed to predict as much metabolic information as possible for a given species, with the idea to curate the predictions afterwards [27]. In order to validate the predictions by computational methods, existing manually curated metabolic networks can be used. The main advantage of these curated metabolic networks is that errors caused by misannotation in protein databases and thereby incorrect or incomplete labeling of metabolic function, have been corrected. Moreover, specific gene function annotations (e.g. transport system components and annotations made by pathway analysis [10]), specific reactions (e.g. not present in KEGG) and potentially protein complex definitions are other promising features of manually curated networks which can be used as a source to predict and/or to validate metabolic networks.

Here, we describe a semi-automatic approach to accelerate the process of genome-scale metabolic network reconstruction by taking full advantage of already manually curated networks. The developed method, referred to as the AUTOGRAPH-method (AUtomatic Transfer by Orthology of Gene Reaction Associations for Pathway Heuristics), is applied to the annotated genome of *Lactococcus lactis *IL1403. To evaluate the AUTOGRAPH-method, we compared predicted gene-reaction associations with a manually curated and published metabolic network of *L. lactis *IL1403, which we will refer to as the Oliveira network [31]. The AUTOGRAPH-method recovered most of the gene-reaction associations (74 – 85%) and we predicted many additional gene-reaction associations. The results illustrate the predictive power of the AUTOGRAPH-method, which may also be applied to unannotated genomes.

## Results

**Figure 1:**
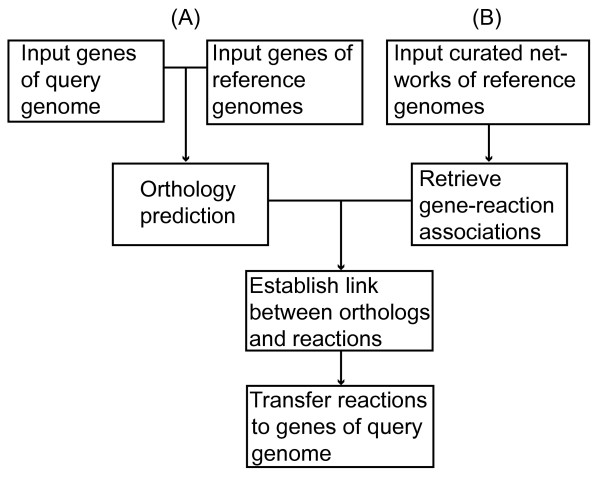
Schematic representation of the AUTOGRAPH-method. The method consists of two parts, A and B. Part A includes the orthology prediction between genes of the query genome and the reference genomes from which there is a manually curated metabolic network available. In part B the gene-reaction associations are extracted from the manually curated networks. Subsequently, orthology will be combined with the gene-reaction association data. This allows reaction transfer to genes of the query genome.

We applied the AUTOGRAPH-method (see Figure 1 and methods for details) to the genome of *Lactococcus lactis *IL1403 [32] using three manually curated metabolic networks as input, i.e. a network from *Escherichia coli *K12 [22], *Lactobacillus plantarum *WCFS1 [manuscript in preparation, see also [17]] and *Bacillus subtilis *[33]. In total, we predicted 525 genes of *L. lactis *to be associated to 416 different reactions (additional file 1). To validate these results we compared the predicted metabolic network with the Oliveira network [31] by examining gene-reaction associations in both networks.

### AUTOGRAPH versus Oliveira network

**Figure 2:**
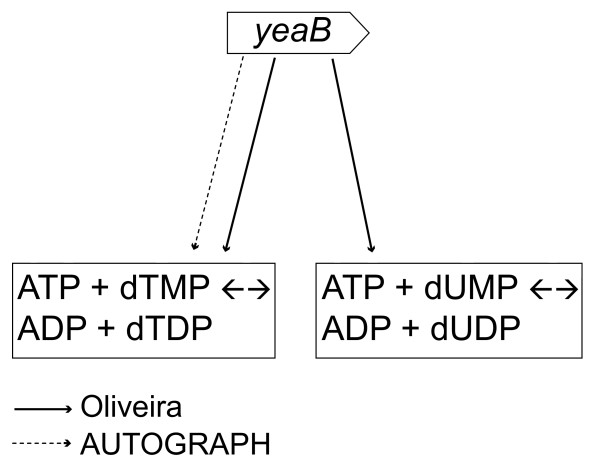
Example of differences in gene-reaction associations between AUTOGRAPH and Oliveira network. Two very similar metabolic reactions (one involving dTMP and the other dUMP) are associated to the gene *yeaB *in the Oliveira network. One of the two gene-reaction associations was not recovered from the predicted network, because the metabolic reaction (i.e. ATP + dUMP ↔ ADP + dUDP) was not associated to the orthologs in *E. coli*, *B. subtilis *and *L. plantarum*.

We could recover 349 genes from the Oliveira network (see methods), involving 464 gene-reaction associations. Gene-reaction associations are one-to-one relationships between genes and metabolic reactions. One single gene can be involved in multiple gene-reaction associations, as certain gene products (i.e. enzymes) are able to catalyze multiple reactions (see Figure 2 for an example). We found that 342 (i.e. 74%) of the Oliveira gene-reaction associations were also predicted by the AUTOGRAPH-method (referred to as confirmed associations in the remaining of the text). 122 were not predicted by AUTOGRAPH. Therefore, we investigated and categorized these associations into three groups: (I) *L. lactis *genes that are organism-specific compared to *E. coli*, *B. subtilis *and *L. plantarum *(in 19% of the 122 cases). As a consequence, it is not possible to predict orthologs and metabolic reactions. (II) Predicted orthologs were lacking reaction associations in the input networks of *E. coli*, *B. subtilis *and *L. plantarum *(in 29% of the 122 cases). Absence of metabolic reactions within the input networks is related to specific choices made by curators during modeling studies. A lack of detailed experimental evidence is one reason to exclude reactions. For example, the aspartate aminotransferase of *L. lactis *(gene name: aspC) is orthologous to yfbQ of *E. coli*, but yfbQ has not been incorporated within the *E. coli *metabolic network due to a lack of experimental evidence (according to the Ecocyc database [16]; note that there is an aspartate aminotransferase in *L. lactis*, gene name: aspB, which has been predicted by AUTOGRAPH). (III) Additional reactions were associated to genes in the Oliveira network compared to the equivalent genes in the input networks (in 52% of the 122 cases, see Figure 2). For example, the *L. lactis *aromatic amino acid aminotransferase (gene name: araT) is an enzyme with broad substrate specificity, which has been experimentally verified [31]. In total, the enzyme is able to catalyze 18 different reactions of which two have been included within the *L. plantarum *source metabolic network (i.e. difference of 16 gene-reaction associations).

**Figure 3:**
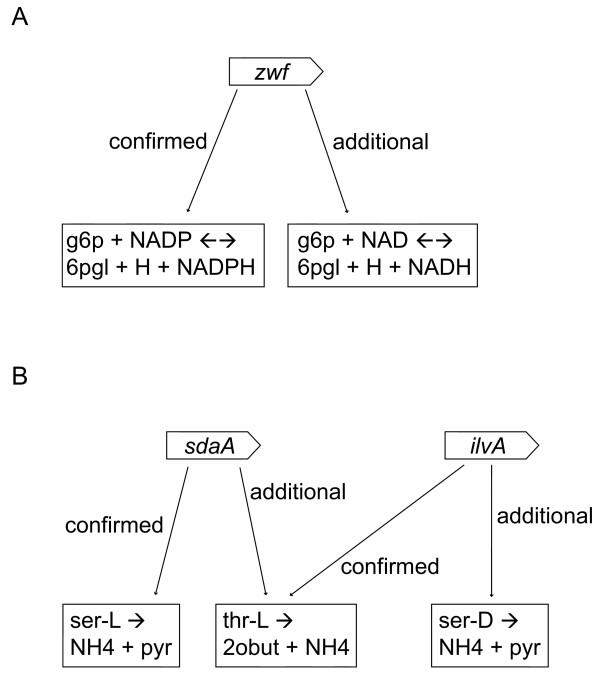
Two different examples of additional gene-reaction associations for genes already present in the Oliveira network. (**A**) Differences in cofactor usage: two predicted gene-reaction associations involving a single *L. lactis *gene (*zwf*), differing on the level of cofactor (i.e. NAD versus NADP). Abbreviations: g6p = D-Glucose 6-phosphate and 6pgl = 6-phospho-D-glucono-1,5-lactone (**B**) Differences in substrate utilization: additional gene-reaction associations were predicted for *sdaA *and *ilvA*, with similar biochemistry but different amino acids as substrate. Note that some of the additional associations involve reactions already associated to other genes (31% of all additional associations). Abbreviations: ser-L = L-Serine, pyr = Pyruvate, thr-L = L-Threonine, 2obut = 2-Oxobutanoate and ser-D = D-Serine.

Besides the 342 confirmed gene-reaction associations, we also predicted 164 additional associations with AUTOGRAPH for the genes that were already present in the Oliveira network. By manually investigation of the additional gene-reaction associations we found that the majority involved metabolic reactions that are highly similar to the reactions within confirmed associations: differences were found on the level of cofactor assignment or a different substrate (see Figure 3 for examples). Nevertheless, the additional reactions with different cofactors are still relevant within network reconstruction, as curators have investigated these in their metabolic modeling studies.

An explanation for the prediction of many additional gene-reaction associations is that we explicitly included recent gene-duplicates in the analysis (see methods). Recent gene-duplicates are likely to catalyze very similar metabolic reactions (i.e. molecular function)[34]. As a consequence, in some cases the reaction associations of two (or more) genes are transferred to a single *L. lactis *gene. It is very well possible that the *L. lactis *genes are able to catalyze the different reactions, but the availability of substrates or cofactors will determine whether or not these reactions will occur. Moreover, we found that 31% of the additional associations involved metabolic reactions that were already present in the Oliveira network, indicating that also a single metabolic reaction can be associated to multiple genes (Figure 3B). Such associations are especially of interest in integrative functional genomics studies (i.e. gene-expression).

**Figure 4:**
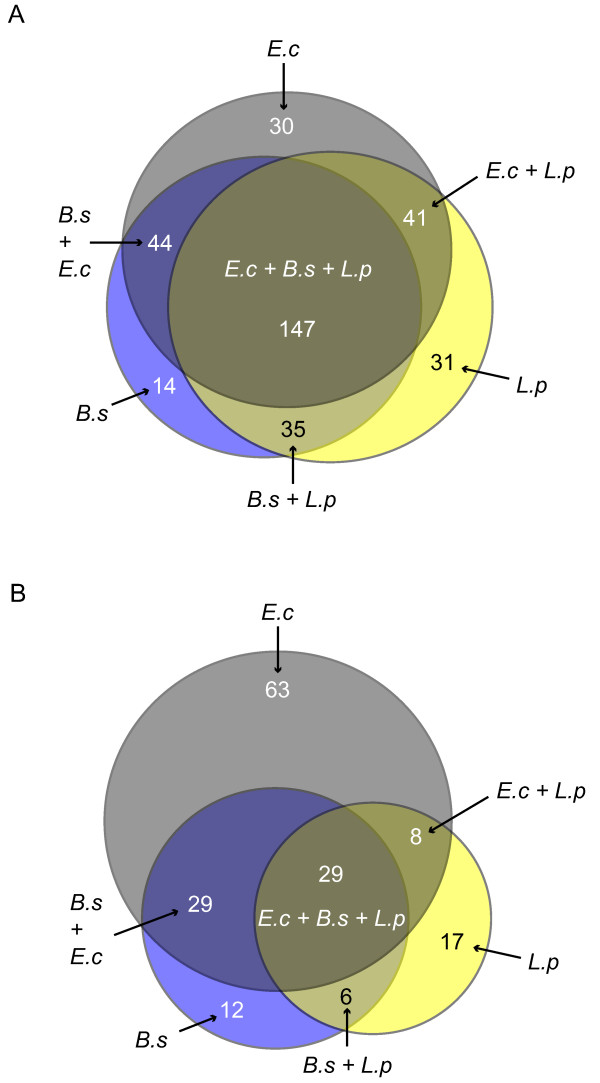
Contribution of the three input metabolic networks of *E. coli *(*E.c*), *B.subtilis *(*B.s*) and *L. plantarum *(*L.p*) to the number of confirmed and additional gene-reaction associations for *L. lactis*. (**A**) Gene-reaction associations confirmed in the Oliveira network. Notice that a substantial number of gene-reaction associations were exclusively derived from a single network (30 of *E.c*, 14 of *B.s *and 31 of *L.p*, respectively). (**B**) Additional gene-reaction associations not present in Oliveira network. A substantial number of gene-reaction associations were exclusively derived from single networks (e.g. 38% were derived from *E.c*).

By examination of the orthologs and their associated metabolic reactions, we observed that all three metabolic networks contributed to the total number of predicted gene-reaction associations (i.e. confirmed and additional, Figure 4). To certain extent the three networks overlap in terms of gene-reaction associations, but we also derived 22% of the confirmed associations from single networks (Figure 4A). This indicates that by increasing the number of metabolic networks for the AUTOGRAPH-method we could positively affect the number of (correct) predicted gene-reaction associations (at least for *L. lactis*). On the other hand, an increase in the number of input metabolic networks also increases the number of additional gene-reaction associations (Figure 4B), causing an increasing need for manual curation (i.e. a decision to incorporate an association).

### Additional predictions for genes not present in Oliveira network

In the comparison between the predicted metabolic network and the Oliveira network, we also found many gene-reaction associations involving genes that were not present in the Oliveira network (see additional file 1). In order to explore the value of these additional predictions, we manually investigated the genes that were associated to reactions. In total, 203 additional genes were found to be associated to 263 different reactions. We categorized the genes into three groups: (I) transport: all genes associated to transport reactions, (II) specific: incorporation of these genes and their associated reactions would potentially lead to additional biochemistry and hence possibly different model predictions and (III) choice: these genes and associated reactions would lead to a higher resolution of the network, i.e. would specify reactions that are lumped into an overall reaction in the Oliveira network. The majority of genes (i.e. 99) were classified into the group of specific. For example, 15 genes were predicted to be associated to vitamin biosynthesis reactions. We analyzed whether the associated metabolic reactions were already present in the Oliveira network or whether the reactions were new and could therefore add new metabolic capabilities to the network. Overall we found 60 new metabolic reactions associated to the 99 investigated genes (see additional file 1). These reactions included potentially important metabolic routes, such as glycogen metabolism and the phosphoketolase reaction. The latter enzyme is involved in pentose catabolism in many lactic acid bacteria (see also additional file 1 for examples of predicted genes and their associated metabolic reactions with literature support) [35].

Of the remaining 104 genes, 53 genes appeared to encode for transport system components and 51 genes were categorized into the group of choice. The group of choice included the genes that will contribute to a higher level of detail to metabolic pathways which already existed (but were lumped) in the Oliveira network. For example, the group of choice included all amino acid tRNA-ligases which were not included in the Oliveira network because protein synthesis was considered as a lumped reaction starting with the individual amino acids as precursors, and protein as product [31]. The genes and the associated metabolic reactions in this group would not alter model predictions, but they are useful extension to the model when it is used for functional genomics data integration and analysis. Thus, the incorporation of this group of genes is a matter of choice and depends on the specification and final purpose of the metabolic network.

### AUTOGRAPH versus Pathologic

As various automatic approaches exist to predict gene-reaction associations for a metabolic network, we also compared the AUTOGRAPH-method to one well established approach called Pathologic, which is part of the Pathway Tools software [27]. The Pathologic algorithm takes annotated genomes as input and predicts gene-reaction associations based on name-matching and EC-codes. The EC-code approach to link metabolic information to genes is similar to other methods, such as IdentiCS and metaSHARK [28,29]. Pathologic is the first step in the construction of a so-called PGDB (pathway-genome database) which consists of gene-reaction associations. Several organism-specific PGDBs have been constructed and manually curated [16,17,36,37]. In order to perform a reliable comparison between the AUTOGRAPH-method and Pathologic, we specifically selected two manually curated PGDBs, i.e. EcoCyc [16]and LacplantCyc [17] as a source to predict gene-reaction associations by orthology for *L. lactis *IL1403. We predicted a total of 580 *L. lactis *genes to be associated to one or multiple reactions. Of these, 394 showed an overlap with the *L. lactis *metabolic network predicted automatically by Pathologic (i.e. 394 genes were present in both predicted networks, the majority of which showed consistency in associated metabolic reactions). Therefore, 186 additional genes were exclusively predicted by AUTOGRAPH to be associated to one or multiple reactions. In contrast, only 35 metabolic genes were exclusively predicted by Pathologic.

The 186 additional predicted genes included specific metabolic genes as well as a relatively high fraction (i.e. 43%) of transport system components. Absence of transporters was to be expected because transporters do not have an EC-code and are therefore not considered by the Pathologic algorithm: transport reactions need to be added manually to the automatically predicted PGDBs. The results demonstrate the strength of the AUTOGRAPH-method being complementary to Pathologic. Both methods can thus be combined to predict organism-specific metabolic networks.

### Metabolic network reconstruction and protein complexes

**Figure 5:**
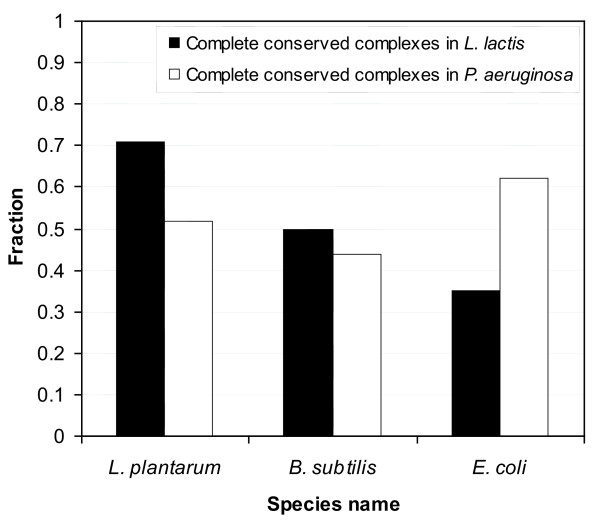
Predicted complete protein complexes for *L. lactis *and *P. aeruginosa*, based on a pairwise comparison with protein complexes found in metabolic networks of *L. plantarum*, *B. subtilis *and *E. coli*. A higher fraction of protein complexes is completely conserved between *L. plantarum*, *B. subtilis *and *L. lactis*, compared to *E. coli *and *L. lactis*. As *L. plantarum*, *B. subtilis *and *L. lactis *are all gram-positives and *E. coli *is a proteobacterium, the phylogenetic relationship between species appears of influence in number of completely conserved protein complexes. This is further supported by applying the AUTOGRAPH-method to the annotated genome of *P. aeruginosa*, which shows that the fraction of (predicted) complete protein complexes is relatively higher between *P. aeruginosa *and *E. coli *(i.e. both are proteobacteria), compared to *P. aeruginosa *and the gram-positive bacteria (*L. plantarum *and *B. subtilis*).

In this study we mainly focused on the automatic prediction of gene-reaction associations for a metabolic network of *L. lactis*. Another factor which is of importance in metabolic network reconstruction, especially in relation to functional genomics data analysis (i.e. proteomics or gene-expression data), is the incorporation of protein complexes. Therefore, we explored the possibilities to predict protein complexes based on the available curated genome-scale metabolic networks which include both protein complexes and detailed metabolic reactions. Methods have been developed to predict biochemical networks on the level of protein interactions (i.e. possible protein complexes), but these lack the detailed metabolic reaction networks [1,38]. However, the relationship between genes, proteins and their metabolic functions can be many-to-many, making the predictions very difficult. We confined our analysis to testing whether or not all components of protein complexes defined in the metabolic networks of *E. coli*, *L. plantarum *and *B. subtilis*, were also encoded on the genome of *L. lactis *(note that protein complex definitions are based on genomic context or experimentally derived protein-protein interaction data). We excluded those protein complexes from the analysis that involved isoenzymes, as the possibilities for complex definition in such cases can be rather large and are often ambiguous. The analysis revealed that a relatively high fraction of protein complexes defined for *L. plantarum *were completely conserved in *L. lactis *(Figure 5), and are therefore reliable candidates to be automatically incorporated in the *L. lactis *metabolic network (notice that we were not able to validate the predictions, due to a lack of protein complex information in the Oliveira network).

To study a possible relationship between the number of predicted (complete) protein complexes and the phylogenetic relationship between considered species, we predicted protein complexes for the annotated genome of *Pseudomonas aeruginosa *PAO1 (Figure 5). In this case, we specifically took *P. aeruginosa *because it is, together with *E. coli*, taxonomically classified as a proteobacterium. As expected, we observed a higher number of conserved (complete) protein complexes between *P. aeruginosa *and *E. coli*, compared to *P. aeruginosa *and the gram-positive bacteria (*L. plantarum *and *B. subtilis*). On the other hand, we observed a higher number of conserved protein complexes between the gram-positive bacteria (*L. plantarum *and *B. subtilis) and L. lactis*, in contrast to *L. lactis *and the more distantly related *E. coli*. These observations suggest a correlation between the number of complete (conserved) protein complexes and the phylogenetic relationship between two species.

## Discussion

We have developed a procedure which exploits the availability of well-curated metabolic networks to predict gene-reaction associations. As we believe that high-quality metabolic reconstructions always need careful manual curation [1,2], the objective of this study was not to develop a method for fully automated reconstructions, but to develop a method that optimally benefits from existing, well-curated reconstructions. We explicitly focused on developing a method to accelerate decision steps for the incorporation of gene-reaction associations in a metabolic network. This can be achieved by presenting for each query gene its orthologs from manually curated metabolic networks (additional file 1). The main advantage of transferring information from curated networks is the fact that unannotated genomes can be used as input and thereby avoiding misannotation caused by errors in protein databases or incomplete labeling (e.g. incomplete EC-codes). Moreover, specific annotations within the metabolic context can be transferred from these networks allowing the interpretation of organism-specific choices such as reaction stoichiometry and cofactors (e.g. NAD or NADP).

Our method is based on evolutionary concepts (orthology definitions) for gene function prediction. Rather than using bidirectional best hits alone, we used Inparanoid for the orthology definition [39]. Inparanoid has a relatively high resolution and is able to predict recent gene duplicates (i.e. inparalogs): these are genes from a single species that are *all *orthologous to one or multiple genes in another species [40]. Inparalogs are likely to have retained the same or very similar molecular function, and are probably functionally differentiated on the biological process level [34]. The orthology definition therefore allows the transfer of metabolic reactions (molecular function) also in the case of gene duplicates.

In our study we derived gene-reaction associations from metabolic networks of *E. coli*, *L. plantarum *and *B. subtilis*, by predicting orthologs between these three species and *L. lactis*. We have shown that the number of confirmed (predicted) gene-reaction associations for *L. lactis*, for the genes that are present in the published Oliveira network, was relatively high (i.e. 74%). The coverage of 74% is calculated with the highest stringency, as we distinguished reactions with different cofactor usage and different (but similar) substrates. Exact cofactor definitions, however, are frequently ignored in studies on the topology of metabolic networks (e.g. functional genomics data analysis) [12]. When ignoring cofactor usage, i.e. considering the reactions that differ only in cofactors as identical, the coverage of our method is much higher (i.e. ~85%). Nevertheless, cofactor utilization is still important in modeling approaches such as constraint-based modeling [1,8,9].

We predicted many additional gene-reaction associations for the genes that were already present in the Oliveira network. As one might consider the additional predictions as over-predictions, we investigated the additional gene-reaction associations in more detail. We found that the majority of the additional associated reactions were mainly (small) variations to the reactions in the Oliveira network (Figure 3). In many cases the reactions slightly differed in terms of substrates and cofactors (e.g. reactions that take place with either ATP or GTP). Therefore, the additional predictions should not directly be considered as an over-prediction, as the level of metabolic detail in a network (e.g. including or excluding cofactors, see above) also depends on the exact purpose.

Importantly, we also predicted 203 genes that were absent from the Oliveira network, of which 34 were annotated as hypothetical. A major fraction of these additional gene-reaction associations involved specific metabolic reactions, which could be useful additions to the metabolic network. Even though the actual choice whether or not these specific gene-reaction associations should be included, might depends on the exact goal of the (modeling) study, new associations are useful information for further improvement of the model. Especially in the context of integrative bioinformatics, any new gene-reaction association allows that specific gene to be studied in its metabolic context, and in that respect the new associations found with the AUTOGRAPH-method should be very useful.

Besides the specific gene-reaction associations we also found many transport system components that are absent from the Oliveira network. Although transporters could also be considered as specific reactions, we have classified them into a separate group, because their substrate specificity is in general difficult to predict based on sequence data alone ([34], C. Francke, unpublished results). Contextual information, such as genome context, can be used in respect to the prediction of gene-reaction associations for transport system components. If this has been done in curated metabolic networks, transfer of function by high-resolution (co-)orthology becomes possible (C. Francke, unpublished results). Although transporters may not in all cases be essential in modeling studies, e.g. when growth on specific substrates is studied, they are important for a complete picture of the metabolic capacity of a species.

In the comparison between AUTOGRAPH and Pathologic, we found a relatively large overlap in terms of genes associated to reactions, but we also established many additional genes, including many transporters. Both methods can be combined to predict gene-reaction associations with high coverage, as both predicted a unique set of additional metabolic genes (186 by AUTOGRAPH and 35 by Pathologic). Besides the prediction of gene-reaction associations, Pathologic also associates genes to pathways using the pathway information from MetaCyc (the collection of metabolic reactions and pathways occurring in *all *included species) [41]. This is one of the major advantages of the approach [27], but it also causes much redundancy, as many variations exist in single pathways (e.g. citric acid cycle in different species). Over-predictions of this type have been removed in many curated organism-specific PGDBs which were initially constructed by Pathologic (see ref [17] for an example). Therefore, when using the AUTOGRAPH approach, redundancy in pathway association can be controlled by the number of curated PGDBs used as input. Increasing the number of input databases (or models) will increase coverage, but also increase the number of possible over-predictions (Figure 4).

When we studied the possibility to predict protein complexes, we found that the number of automatically predicted (complete) protein complexes depends on the phylogenetic relationship between species, although it should be noted that we may not have sufficient species to establish a strong correlation (Figure 5). We also observed complexes for which not all constituent proteins were found on the genome of *L. lactis*. The reason for the prediction of these incomplete protein complexes is likely to be the result of (physiological) variations in complexes for the different species (i.e. organism-specific complexes) or a lack in orthology detection. The incomplete protein complexes therefore need to be manually curated to investigate if 'missing' components are absent due to a lack in orthology detection or that components are absent due to physiological differences between the species. Also, specific choices from curators can lead to differences in complex definition, which consequently could influence complex prediction. The presented AUTOGRAPH-method is a possible first step to automate the prediction of protein complexes integrated with a detailed metabolic reaction network.

## Conclusion

We have described a method to accelerate genome-scale metabolic network reconstruction by using orthology and existing manually curated metabolic networks to predict gene-reaction associations. In this study we focused on the prediction of a metabolic network for *L. lactis *IL1403. We recovered most of the gene-reaction associations (i.e. 74 – 85%) that were present in a published metabolic network of *L. lactis *IL1403 (Oliveira network). Moreover, we identified over 200 additional genes associated to reactions that are potentially relevant for either metabolic modeling or integrative bioinformatics. We are, however, aware of the fact that the quality of a metabolic network derived from our developed method depends on the availability and quality of manually curated metabolic networks, and on the orthology detection. This, however, also holds for the annotation in protein databases. We developed the method with the goal to accelerate the process of metabolic reconstruction, by minimizing the adjustments needed during curation, and by giving the curator an overview of the decisions made previously by other curators. We expect that in the future it will become possible to select a substantial number of species which are closely related to (any) query species, because there is a rapid increase in the number of reconstructed genome-scale metabolic networks by ongoing functional genomics projects.

## Methods

### Collection of manually curated genome-scale metabolic networks

We used three manually curated metabolic networks, that of *Escherichia coli *K12 [22], *Lactobacillus plantarum *WCFS1 [B. Teusink *et al*., manuscript in preparation, see also [17]] and *Bacillus subtilis *subsp. subtilis str. 168 [33], as a source to predict automatically a metabolic network for *Lactococcus lactis *IL1403. The developed method is called AUTOGRAPH (AUtomatic Transfer by Orthology of Gene Reaction Associations for Pathway Heuristics, see Figure 1) and is outlined in detail below. The curated networks were initially constructed with Genomatica's Simpheny™ software for constraint-based modeling purposes, and were retrieved as flat-files containing gene-protein-reaction associations [42].

A reference metabolic network of *L. lactis *IL1403 has been used to evaluate our method (discussed below). This network was also constructed for constraint-based modeling purposes and was retrieved from the authors as a flat-file, containing gene-reaction associations. Throughout the article we will refer to this published reference network as the Oliveira network [31].

To compare the automatic reconstruction of *L. lactis *IL1403 metabolic network by Pathologic with that of our method, we used the Genbank NCBI annotation file of *L. lactis *IL1403 as input for the Pathologic software [27,43]. In addition, the same Genbank file together with two manually curated networks from the BioCyc collection (i.e. EcoCyc [16] and LacplantCyc [17]) were used as inputs for our method (discussed below).

### AUTOGRAPH: prediction of orthology and gene-reaction associations

The AUTOGRAPH-method combines manually curated genome-scale metabolic networks and orthology to predict a network for a query species (Figure 1). First, we established (pairwise) orthologous relationships between the genes of *L. lactis *(i.e. query species) and the genomes of *E. coli, B. subtilis *and *L. plantarum *(see additional file 1). The complete genome sequences of *L. lactis*, *E. coli, B. subtilis *and *L. plantarum *were retrieved from Genbank NCBI. The algorithm of Inparanoid, which requires the genome sequences as inputs, has been applied to predict orthologs [39]. The Inparanoid method is based on Bidirectional Best Hits (BBH) and predicts, besides one-to-one orthology (BBH), also inparalogous genes (also referred to as recent gene duplicates). Inparalogs arise from a gene duplication event after a speciation and are therefore orthologous to one or multiple genes from another genome [40]. We applied Inparanoid with the default settings.

Second, the *L. lactis *genes and their corresponding orthologs in *E. coli*, *L. plantarum and B.subtilis *were retrieved from the Inparanoid outputs using the Python programming language [44]. Subsequently, the orthologs were automatically analyzed for associations with one or multiple reactions (gene-reaction associations were derived, using Python, from the flat-files of the curated metabolic networks). In this way, a complete list of all *L. lactis *genes, and their orthologs with associated reactions, was generated automatically (see additional file 1). Such an approach allows for a quick analysis of gene-reaction associations made by other curators and thereby the reconstruction of genome-scale metabolic networks for query species. The method has not been developed as a fully automatic tool, including database construction and visualization techniques, but rather as a frame-work to predict gene-reaction associations in a (semi-)automatic way. Other software exists to visualize and analyze metabolic networks [27,42,45,46].

### Evaluation of AUTOGRAPH

To evaluate the AUTOGRAPH-method we investigated gene-reaction associations in the predicted and the Oliveira network. First, the gene names from the Oliveira network were automatically mapped onto the gene names in the Genbank file of *L. lactis *IL1403 (Genbank files are the input for AUTOGRAPH). In this way, we could recover 349 genes from the Oliveira network (of the 358 reported in the article). Secondly, the comparison of gene-reaction associations has been done manually, as the reaction and compound abbreviations in the Oliveira network and in the input networks for the AUTOGRAPH-method were not identical.

## Authors' contributions

RN and BT have written the manuscript, have developed the AUTOGRAPH-method and analyzed obtained data. FE contributed to the development of AUTOGRAPH. CF and RS both contributed throughout the development of the method by discussions, and by revising the manuscript. All authors have read and improved the final manuscript.

## Supplementary Material

Additional File 1 Additional file 1 has been uploaded as a Microsoft Excel file containing eight worksheets: 1. *Orthology prediction*: List of orthologous gene pairs between *L. lactis*, *E. coli, B. subtilis *and *L*. plantarum. Orthologs were predicted by applying the Inparanoid algorithm, using default settings. 2. *Overlap AUTOGRAPH and Oliveira*: Orthologous groups of genes from the Oliveira network and their orthologs with associated reactions. The reactions associated to the *L. lactis *genes in the Oliveira network are indicated under "Oliveira". 3. *Unique genes for AUTOGRAPH*: Orthologous groups of genes containing the *L. lactis *query genes (from Genbank) which are *not *incorporated into the Oliveira network, and their orthologs with associated reactions. *L. lactis *genes were classified into three different groups: (i) transport: all genes associated to transport reactions (ii) specific: incorporation of these genes and their associated reactions would potentially lead to additional biochemistry and hence possibly different model predictions and (iii) choice: these genes and associated reactions would lead to a higher resolution of the network, i.e. would specify reactions that are lumped into an overall reaction in the Oliveira network. 4. *Unique specific reactions*: List of unique specific reactions that are associated to *L. lactis *genes which are *not *incorporated into the Oliveira network. 5. *Examples and literature support*: Examples of predicted orthologs with literature support for *L. lactis *genes which are not incorporated into the Oliveira network. 6. *Abbreviations E. coli*: Compound information (including compound abbreviations) of reactions which are associated to *E. coli *genes. 7. *Abbreviations B. subtilis*: Compound information (including compound abbreviations) of reactions which are associated to *B. subtilis *genes. 8. *Abbreviations L. plantarum*: Compound information (including compound abbreviations) of reactions which are associated to *L. plantarum *genes. Click here for file
